# Clinical validation of three cardiovascular magnetic resonance techniques to measure strain and torsion in patients with suspected coronary artery disease

**DOI:** 10.1186/s12968-020-00684-2

**Published:** 2020-12-07

**Authors:** Johan Kihlberg, Vikas Gupta, Henrik Haraldsson, Andreas Sigfridsson, Sebastian I. Sarvari, Tino Ebbers, Jan E. Engvall

**Affiliations:** 1 Department of Radiology in Linköping, and Department of Health, Medicine and Caring Sciences, Linköping University, Linköping, Sweden; 2 Center for Medical Image Science and Visualization (CMIV), Linköping University, Linköping, Sweden; 3 Department of Health, Medicine and Caring Sciences, Linköping University, Linköping, Sweden; 4 Department of Radiology and Biomedical Imaging, University of California San Francisco, San Francisco, USA; 5 Department of Clinical Physiology in Linköping, and Department of Health, Medicine and Caring Sciences, Linköping University, Linköping, Sweden; 6 Department of Clinical Physiology & Molecular Medicine and Surgery, Karolinska Institutet, Karolinska University Hospital, 17176 Stockholm, Sweden; 7 Department of Cardiology, Oslo University Hospital, Rikshospitalet, 0316 Oslo, Norway

**Keywords:** Strain, Torsion, Myocardial infarction, DENSE, Tagging, Feature tracking

## Abstract

**Background:**

Several cardiovascular magnetic resonance (CMR) techniques can measure myocardial strain and torsion with high accuracy. The purpose of this study was to compare displacement encoding with stimulated echoes (DENSE), tagging and feature tracking (FT) for measuring circumferential and radial myocardial strain and myocardial torsion in order to assess myocardial function and infarct scar burden both at a global and at a segmental level.

**Method:**

116 patients with a high likelihood of coronary artery disease (European SCORE > 15%) underwent CMR examination including cine images, tagging, DENSE and late gadolinium enhancement (LGE) in the short axis direction. In total, 97 patients had signs of myocardial disease and 19 had no abnormalities in terms of left ventricular (LV) wall mass index, LV ejection fraction, wall motion, LGE or a history of myocardial infarction. Thirty-four patients had myocardial infarct scar with a transmural LGE extent (transmurality) that exceeded 50% of the wall thickness in at least one segment. Global circumferential strain (GCS) and global radial strain (GRS) was analyzed using FT of cine loops, deformation of tag lines or DENSE displacement.

**Results:**

DENSE and tagging both showed high sensitivity (82% and 71%) at a specificity of 80% for the detection of segments with > 50% LGE transmurality, and receiver operating characteristics (ROC) analysis showed significantly higher area under the curve-values (AUC) for DENSE (0.87) than for tagging (0.83, p < 0.001) and FT (0.66, p = 0.003). GCS correlated with global LGE when determined with DENSE (r = 0.41), tagging (r = 0.37) and FT (r = 0.15). GRS had a low but significant negative correlation with LGE; DENSE r = − 0.10, FT r = − 0.07 and tagging r = − 0.16. Torsion from DENSE and tagging had a weak correlation (− 0.20 and − 0.22 respectively) with global LGE.

**Conclusion:**

Circumferential strain from DENSE detected segments with > 50% scar with a higher AUC than strain determined from tagging and FT at a segmental level. GCS and torsion computed from DENSE and tagging showed similar correlation with global scar size, while when computed from FT, the correlation was lower.

## Introduction

Abnormalities in left ventricular (LV) systolic function—whether due to myocardial scarring, ischemia, or electrical conduction delay—are invariably manifested as temporal or spatial changes in myocardial deformation. In routine clinical practice, those abnormalities are primarily assessed visually from cardiac cine-loops acquired by cardiovascular magnetic resonance (CMR) or echocardiography. Such assessment is subjective and requires extensive experience on the part of the observer [1]. In that context, ejection fraction (EF) is the gold standard. However, the concept of strain may be valuable to quantify global as well as segmental shortening and lengthening of the myocardium [2], while torsion, here defined as the difference in rotation between two short axis slices divided by the distance in-between [3], is a promising quantitative measure of global LV function.

Several CMR acquisition techniques may be utilized to calculate strain and torsion: phase contrast [4], tagging [5], displacement encoding with stimulated echoes (DENSE) [6] and feature tracking (FT) of cine-loops [7].

FT uses cine-loops from balanced steady-state free precession imaging (bSSFP), which is part of a standard CMR study and requires little user interaction to perform.

Myocardial tagging can be acquired in two or three dimensions. Most often, grid lines in a rectangular pattern are imposed on the short-axis (SAx) or long-axis (LAx) view of the LV at the R-peak of the electrocardiogram (ECG). The saturation grid fades due to T1-relaxation, but usually lasts until mid-diastole. Tagging is considered the gold standard for CMR deformation imaging [8]. DENSE, introduced in the late 1990s, is a technique which has similarities with tagging [6]. Both techniques have been validated in phantom studies [9, 10]. DENSE measures the tissue displacement using the phase of the CMR signal. DENSE offers several advantages; such as high spatial resolution and relatively simple post processing [8].

A head-to-head comparison of all three techniques in determining abnormal myocardial deformation in coronary artery disease (CAD), has so far been lacking.

We aimed to compare global strain and torsion assessed by DENSE, tagging and FT in relation to clinical parameters such as LV volumes, wall motion and EF, and, secondly, to determine the sensitivity and specificity for segmental strain derived from DENSE, tagging and FT for the detection of myocardial scar with a transmurality in excess of 50%.

## Methods

### Study population

125 patients, all participants in the Doppler-CIP study [11] at Linköping University Hospital, were prospectively enrolled from November 2010 until March 2012. Nine patients were excluded from the analysis due to inadequate image quality—one in DENSE, three in tagging, one in both DENSE and tagging and four in FT, resulting in data from 116 patients available for analysis, 89 (77%) males with a mean age of 67 years (range 49–85). Inclusion criteria were: (1) a history of typical angina or high risk of CAD (> 15% risk of developing cardiovascular events according to the European risk SCORE at the time of assessment [12]), or a positive stress test with > 2 mm ST-depression, (2) known CAD, defined as prior myocardial infarction (> 3 months), or CAD on invasive coronary angiogram. Exclusion criteria were; unwillingness to participate, an acute coronary syndrome during the preceding three months, more than moderate valvular disease, pacemaker implantation, claustrophobia, an estimated glomerular filtration rate < 60 ml/min/1.73 m^2^ or permanent atrial fibrillation. The Doppler-CIP study used a wide inclusion criterion. Therefore, the patient cohort was divided into a group without imaging signs of cardiac disease, having normal LV mass (LVM), blood pressure, LVEF, and wall motion and no signs of late gadolinium enhancement (LGE), and a group with imaging signs of cardiac disease. In addition, a group of 10 healthy subjects was recruited. They declared a history free from cardiovascular disease, did not take cardioactive drugs and had a normal transthoracic echocardiographic exam. These healthy subjects underwent a limited investigation including cine for volumes, FT strain and a specific acquisition for DENSE strain.

### Image acquisition

We acquired CMR data using a 1.5 T CMR system (Achieva Nova Dual, Philips Healthcare, Best the Netherlands) with a protocol including cine bSSFP, tagged images, DENSE, and LGE, all acquired during breath holding. The cine bSSFP images were acquired in the LAx views (2-, 3-, and 4-chamber views) as well as in a stack of SAx views covering the entire LV. Typical parameters were: slice thickness 8 mm, TR/TE 3.6/1.81, flip angle 60°, field of view 350 × 350 mm, matrix 288 × 288 and breath holding time 16 s. The cine images were retrospectively reconstructed into 30 cardiac phases, corresponding to a temporal resolution in the range of 24–41 ms (mean 37 ms).

Tagged images were acquired using complementary spatial modulation of magnetization (CSPAMM), with three SAx planes (basal, mid, and apical) planned in end systole (ES). The typical acquisition parameters for tagging were: inter-tag distance 8 mm, slice thickness 6 mm, TR/TE 4.6/2.1 ms, flip angle 15°, field of view 320 × 320 mm, matrix 256 × 256, 21 heart phases, and breath holding time 16 s. The mean temporal resolution was 43 ms.

DENSE images were acquired as previously described [13] in the same three SAx positions as the tagged images. Briefly, the DENSE sequence is built on three balanced multipoint encodings and threefold SPAMM (3-SPAMM) with fat suppression achieved by using a water-selective first radiofrequency pulse in the DENSE 1-1 SPAMM preparation. A six-shot spiral acquisition was used, with spiral readout interleave duration of 8 ms and TR/TE 11.2/1.27 ms, respectively and three interleaves were acquired each cardiac cycle. A through-slice dephasing of 0.25 Hz was used, and an in-plane displacement encoding strength of 0.30 Hz/pixel. We acquired three time frames: 45 ms prior to aortic valve closure, at valve closure, and 45 ms after the valve closed—where the time of valve closure was determined by bSSFP in the apical LAx view. The acquisition duration was 18 heartbeats, and the spatial resolution was 1.36 mm × 1.36 mm with a slice thickness of 6.0 mm.

LGE data were acquired at the same slice positions as the cine images (three LAx views and a stack of SAx slices) beginning on average 14 min (11–23 min) after the administration of 0.2 mmol/kg bodyweight gadopentetate dimeglumine (Bayer Healthcare, Berlin, Germany). An inversion recovery 3D spoiled gradient echo sequence with TR/TE 4.4/1.3 ms, respectively, was used. Slice thickness and slice gap in the SAx direction were 10 mm and -5 mm (5 mm overlap), respectively.

### Image analysis

FT was performed on the SAx cine bSSFP stack and was limited to Lagrangian analysis, where the comparison was focused on Lagrangian circumferential strain. The LV was divided into 16 segments, excluding the apical cap from the 17-segment model of the American Heart Association [14]. The observer was blinded from the patient history and other image sequences when analyzing each image type.

A single observer (#1) extracted LV volumes, LVEF, and LVM from the SAx cine images using Segment v 1.9 R2966. Wall motion was determined from all cine views (three LAx and one stack of SAx) using the following qualitative scores: 1 = normokinesis; 2 = hypokinesis; 3 = akinesis; 4 = dyskinesis.

A single observer (#2) performed the FT analysis using the 2D-CPA MR software (v 1.2, TomTec Imaging System, Unterschleissheim, Germany). The endocardium and the epicardium were delineated manually in diastole and the software tracked the displacement of 48 points along the endocardium. Lagrangian strain was measured for both the endocardial and the epicardial layers, the mean of which provided transmural strain [15, 16]. The rotation of the apical and basal slice were computed in the software, then divided manually by the slice distance to obtain torsion.

The tagged images were segmented at a later time by observer #2 using Segment (v 2.2 R7056, Medviso AB, Lund, Sweden). Sixty points along the epicardial circumference and 30 points along the endocardium of each slice were semi-automatically tracked to compute Lagrangian strain from the deformation of the line connecting the points [17]. Torsion was obtained as the difference in rotation between two slices, as with FT.

Two observers (#3 and 4) segmented the myocardium for analysis of the DENSE data in the circumferential direction. The endocardium and the epicardium were delineated manually, and the slices were semi-automatically divided into segments, with reference to the attachment of the right ventricular (RV) anterior wall to the septum. We analyzed transmural Lagrangian strain for 16 segments using a DENSE analysis framework developed in-house using MATLAB (R2010b, Mathworks, Natick, Massachusetts, USA). Images with abnormal phase wrapping were automatically excluded. Reproducibility has been previously reported [13].

One observer (#1) determined positive LGE on both a segmental level expressed as mean scar thickness, “transmurality”, and on a global level as a percentage of LVM (Segment v 1.9 R2966, Medviso AB, Lund, Sweden). The software suggests the delineation of scar using a weighted method that was manually corrected if needed. The weighted method involves the following steps: 1. The mean signal intensity and standard deviation are calculated in five sectors in each section. The midmural half of the sector with the lowest mean signal intensity is considered remote myocardium. 2. A section-specific threshold level is calculated by adding the mean of the remote sector and a fixed number of standard deviations from the mean signal intensity in the remote region. 3. A fast-level set algorithm is applied, and the speed term is set to a value calculated by subtracting the section-specific threshold level from the signal intensity [18].

### Reference values

In this study, LVEF was considered reduced when below 57% and the normal reference span for LVM index (LVMI) was 45–81 g/m^2^ for males and 37–77 g/m^2^ for females, as recommended when papillary muscles are excluded [19]. The presence of hypertension was determined when systolic and diastolic blood pressure exceeded 140/90 mmHg respectively, according to prevailing recommendations in 2010 [20].

### Comparison methodology

The evaluation included two key components: (1) a global analysis of torsion and strain amplitude [Global circumferential strain (GCS), global radial strain (GRS)] compared to LVEF, LV volumes, LVMI, and LGE, (2) a segmental analysis of strain to detect segments with LGE transmurality > 50% using area under the curve (AUC) in a receiver operating characteristics (ROC) curve analysis.

### Statistical analysis

The statistical analyses were performed using SPSS (Statistical Package for the Social Sciences, International Business Machines, Inc., Armonk, New York, USA). Analysis of skewness and kurtosis showed normal distribution, permitting Pearson correlation, Student’s t-test and Linear regression to be used. We calculated the sensitivity at 80% specificity from the ROC curve analysis. The level of significance was set to either **0.01 or *0.05.

## Result

### Patient characteristics

**Fig. 1:**
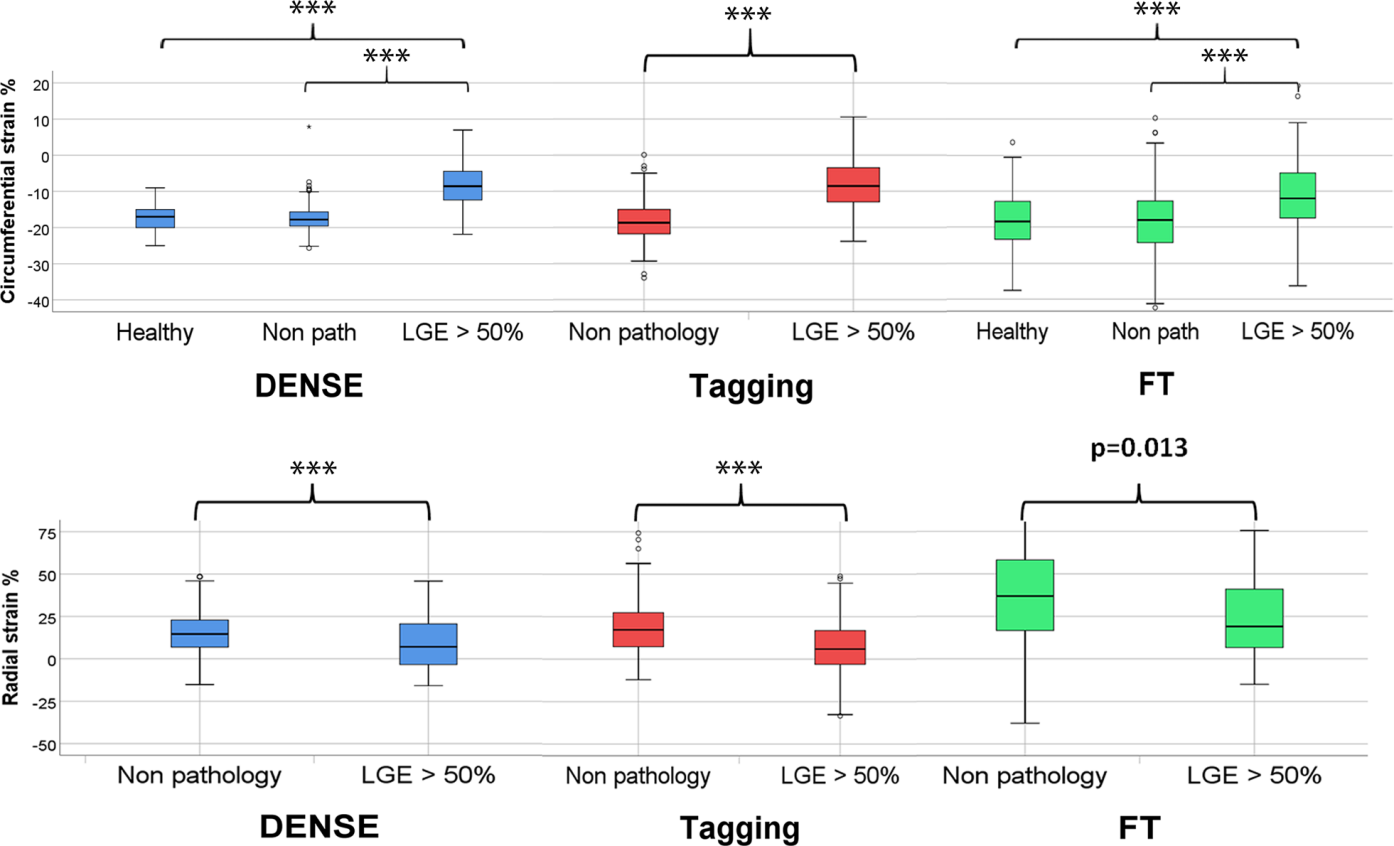
Strain in segments with scar transmurality > 50%. Boxplot of segmental circumferential strain (top row) and radial strain (lower row) assessed using three techniques in ten healthy subjects (160 segments) and patients with no signs of pathology in terms of left ventricular (LV) wall mass index, systolic blood pressure (SBP), diastolic blood pressure (DBP), wall motion, late gadolinium enhancement (LGE) and a history of myocardial infarction, (“Non pathology”, 304 segments) and segments with LGE in excess of 50% wall thickness (LGE > 50%, 84 segments). The box plots show median, the two central quartiles in the box, one quartile in each whisker and outliers. Student’s t-test showed significant differences between the two patient groups for all three techniques.***p < 0.001. Healthy subjects depicted for comparison

**Fig. 2:**
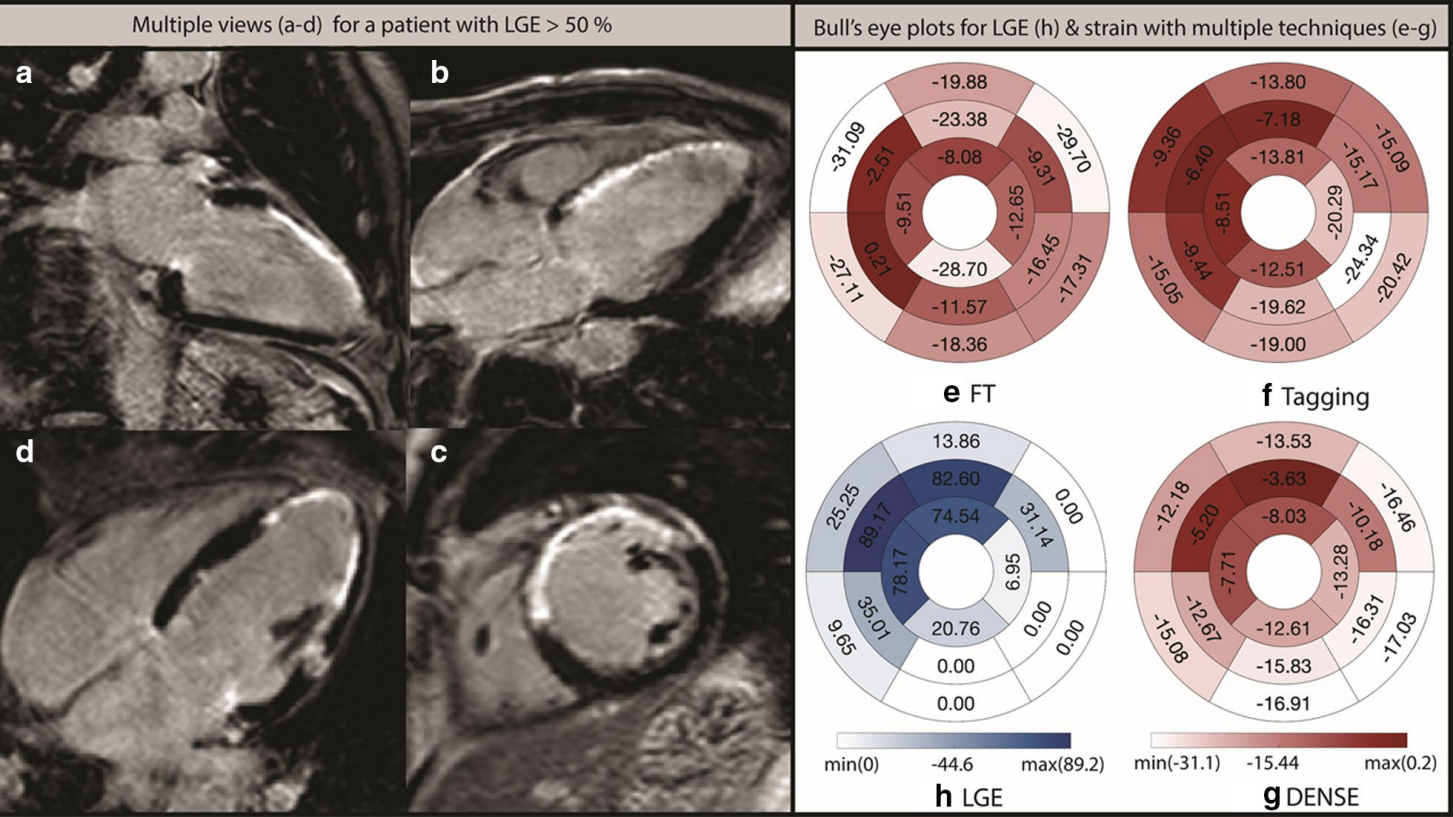
Anteroseptal infarct scar. Images and measurements in a 68-year-old male with extensive anteroseptal and apical infarct scar with transmurality > 50%. Scar volume was 25% of the LV myocardial mass, and the LV ejection fraction (EF) was 42%. The left-hand panel LGE images (**a**–**d**) show the extent of scar. The right-hand panel bull’s eye (**h**) shows percentages for segmental transmurality. The remaining panels show circumferential strain assessed by (**e**) FT, (**f**) Tagging, (**g**) DENSE

Patients were separated into those with signs of myocardial disease (n = 97, 1552 segments) and those free from manifestations of myocardial disease (n = 19, 304 segments) determined from LVMI, LVEF, the presence of abnormal wall motion, positive LGE or a history of myocardial infarction (Table 1). The ten healthy subjects were on average 13 years younger than the patients, had similar LVMI but larger LV volumes. As expected, patients with myocardial disease had larger LV end-systolic volume than the reference group (p = 0.037) and their LVEF trended lower (p = 0.014). Thirty-four patients (84 segments) had LGE in excess of 50% wall thickness in at least one segment, and 37 patients had an LVEF below 57%. Patients with signs of myocardial disease had lower DENSE circumferential strain amplitude and higher standard deviation than those without myocardial disease manifestations. There is a significant difference in strain for segments in the group with no sign of myocardial disease compared to segments with > 50% LGE extent for all three techniques. The overlap between the segment groups was greater with FT than with the other techniques (Fig. 1). Figure 2 shows an example of a patient with a large infarct scar, with circumferential strain assessed by DENSE, FT and tagging in the corresponding segments.

**Table 1:** Patient characteristics

Parameter	Healthy subjects (n = 10)	No manifestations of disease (n = 19)	Manifestations of disease (n = 97)	p-value (no manifestations vs manifestations of disease)
Age (years)	52 ± 4	65 ± 8	67 ± 7	ns
BMI (kg/m^2^)	22 ± 2	26 ± 3	27 ± 3	ns
Male (%)	40	63 ± 0.5	79 ± 0.4	ns
LVMI_at ED_ (g × m^−2^)	51 ± 15	51 ± 9	58 ± 13	ns
Ejection Fraction (%)	61 ± 5	66 ± 6	58 ± 12	0.014
SBP (mmHg)	133 ± 13	128 ± 8	139 ± 12	< 0.001
DBP (mmHg)	80 ± 10	76 ± 7	81 ± 10	ns
LVEDV (mL)	168 ± 53	139 ± 32	158 ± 37	ns
LVESV (mL)	67 ± 27	49 ± 16	67 ± 31	0.037
MI < 1 year (%)	0	0	12	< 0.001
PCI (%)	0	0	9	0.003
Global LGE (%)	0	0	5 ± 7	< 0.001

Data from ten healthy subjects (left) and from the coronary artery disease population, which is divided into one group of patients with imaging signs of disease (n = 97, right) and one lacking imaging signs of disease (n = 19, middle) in terms of left ventricular wall mass index (LVMI), systolic blood pressure (SBP), diastolic ejection fraction (DSP), wall motion, late gadolinium enhancement and a history of myocardial infarction. *BMI* body mass index, *DBP* diastolic blood pressure, *LVEDV* left ventricle end diastolic volume, *LVESV* left ventricle end systolic volume, *MI* myocardial infarction, *PCI* percutaneous coronary intervention, *LGE* late gadolinium enhancement, global percentage. Mean with standard deviation

### Global strain and torsion compared to clinical data

**Fig. 3:**
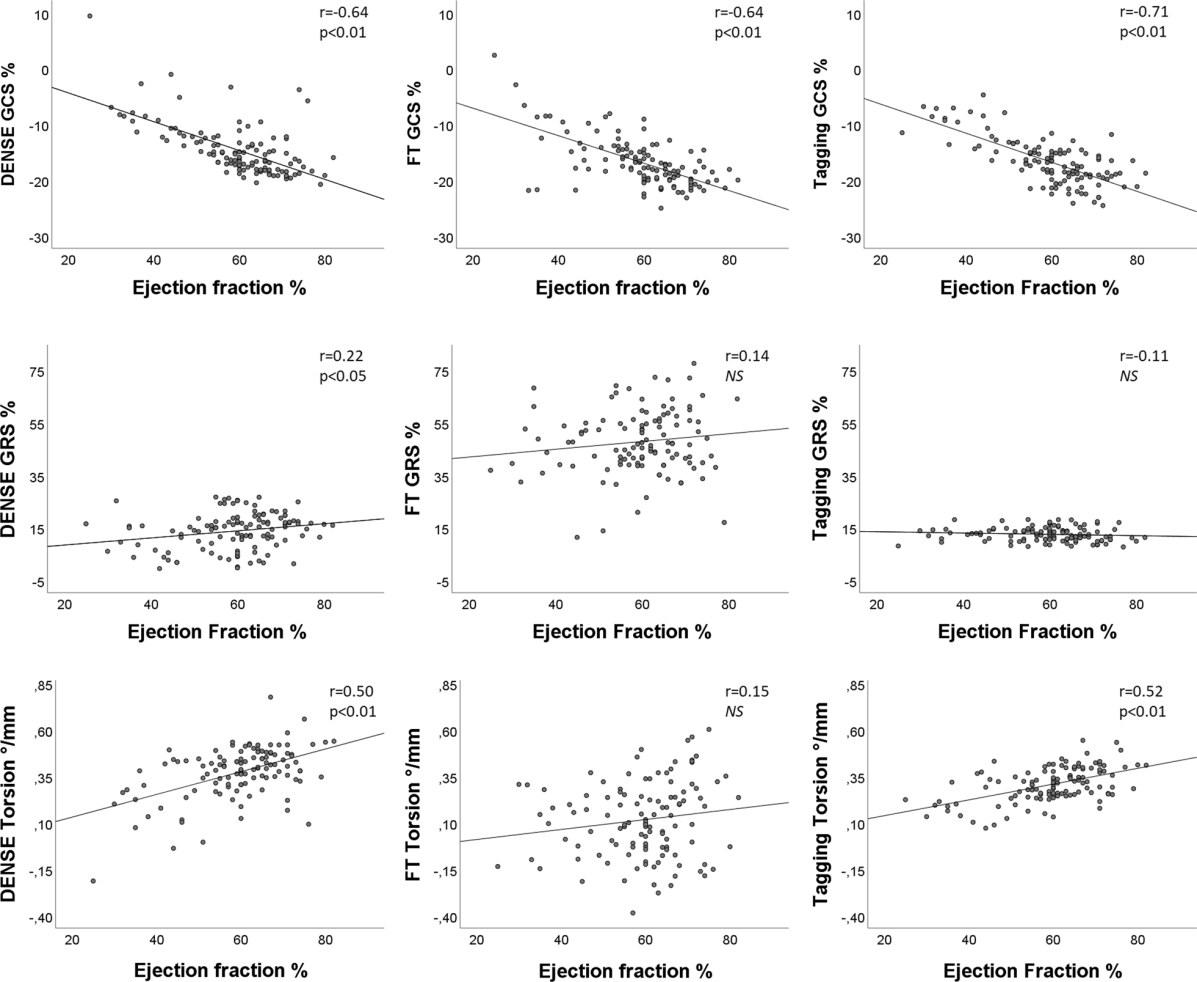
Correlation of global strain and torsion with left ventricular ejection fraction. Pearson correlations (r) between global circumferential strain (GCS, top row), global radial strain (GRS, middle row) and torsion (lower row) and left ventricular ejection fraction. GCR, GRS and torsion assessed by DENSE, FT, and tagging for 116 patients plotted against ejection fraction. The best-fit linear regression as well as the Pearson correlation coefficient and its p-value are shown for each plot. *NS* no significant correlation

**Fig. 4:**
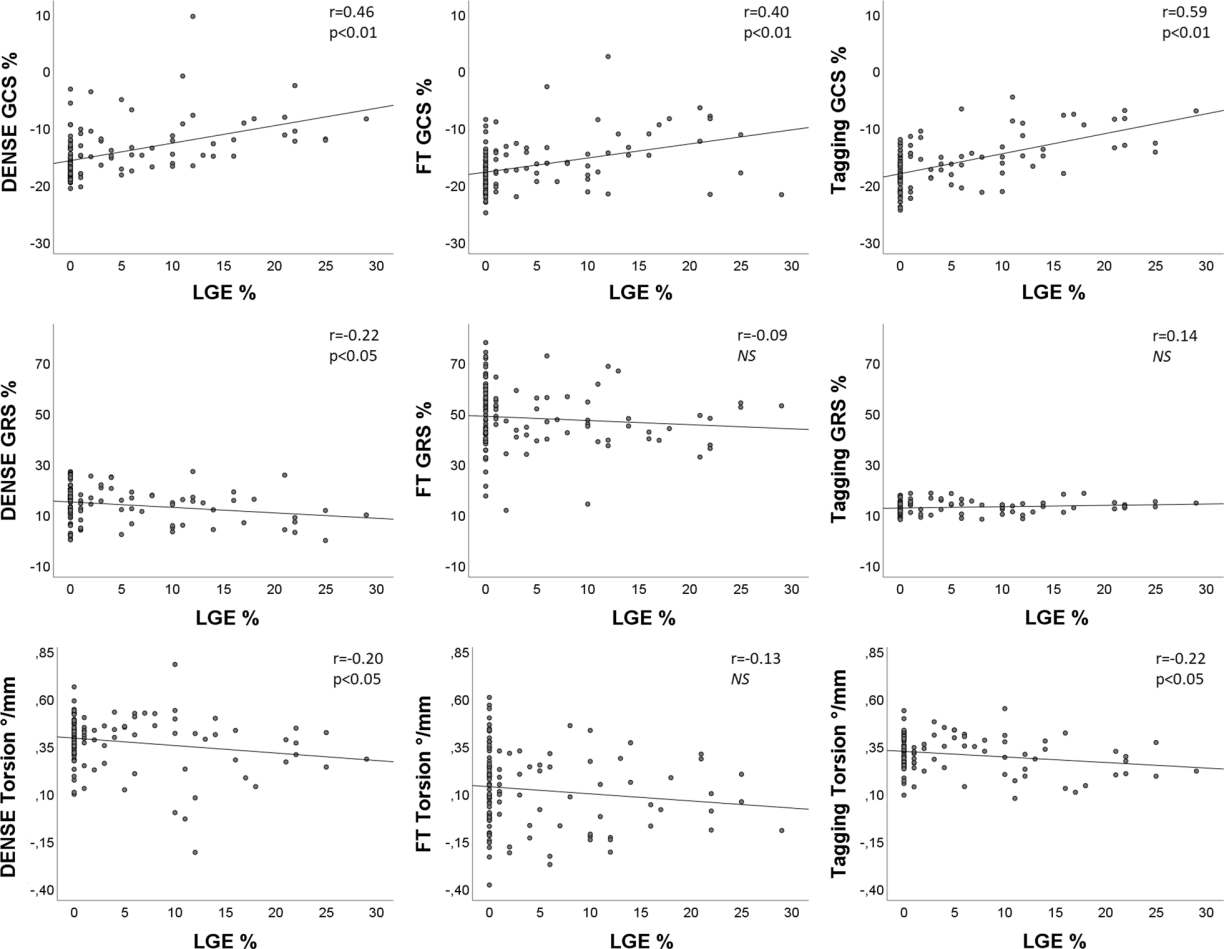
Correlation of global strain and torsion with late gadolinium enhancement (LGE). Pearson correlations (r) between global circumferential strain (GCS, top row), global radial strain (GRS, middle row) and torsion (lower row) vs global LGE as a percentage of the total wall mass. GCR, GRS and torsion assessed by DENSE, FT, and tagging for 116 patients plotted against LGE. The best-fit linear regression as well as the Pearson correlation coefficient and its p-value are shown for each plot. *NS* no significant correlation. The best correlation is seen between tagging strain amplitude and LGE while no correlation is seen between torsion and LGE

GCS was for patients with no signs of myocardial disease (n = 19) − 16.1 ± 3.7% (DENSE), − 18.3 ± 1.6% (FT) and − 18.6 ± 2.8% (tagging). In the same group of patients torsion was 0.37 ± 0.09°/mm (DENSE), 0.12 ± 0.26°/mm (FT) and 0.31 ± 0.08°/mm (tagging). GCS, GRS and torsion results were correlated with other global parameters such as LVEF, blood pressure, LV volumes, LGE and LVMI, see Table 2. The correlations between GCS and GRS vs LVEF were r = 0.64 or r = 0.71 shown in Fig. 3. The correlation between GCS, GRS, torsion, and the global percentage of scar was low, but higher for GCS (Fig. 4). The best-fit linear regression between strain and scar size was for tagging GCS vs LGE r = 0.52 while DENSE GCS vs LGE was r = 0.46.

**Table 2:** Correlations between global circumferential strain (GCS), global radial strain (GRS) and torsion with other global parameters in 116 patients

n = 116	LVEF	SBP	LVEDV	LVESV	LGE	LVMI
DENSE GCS	− 0.635**	− 0.289**	0.301**	0.533**	0.461**	0.316**
FT GCS	− 0.635**	− 0.324**	0.233*	0.438**	0.389**	0.248**
Tagging GCS	− 0.714**	− 0.264**	0.406**	0.666**	0.586**	0.315**
DENSE GRS	0.223**	− 0.046	− 0.219*	− 0.257**	− 0.217*	− 0.126
FT GRS	0.138	0.158	0.075	− 0.013	− 0.94	0.086
Tagging GRS	− 0.110	− 0.072	0.184*	0.141	0.070	0.173
DENSE torsion	0.498**	0.179	− 0.394**	− 0.501**	− 0.196*	− 0.242**
FT torsion	0.150	0.086	− 0.178	− 0.154	− 0.128	− 0.258**
Tagging torsion	0.519**	0.138	− 0.415**	− 0.541**	− 0.218*	− 0.219*

Correlations between global circumferential strain (GCS), global radial strain (GRS) and torsion with other global parameters in 116 patients. Strain and torsion derived from DENSE, FT and tagging with Pearson correlation with left ventricular ejection fraction (EF), systolic blood pressure (SBP), left ventricle end diastolic volume (LVEDV), left ventricle end systolic volume (LVESV), late gadolinium enhancement (LGE) and left ventricle mass index (LVMI). **Correlation is significant at the 0.01 level and *at the 0.05 level

### Segmental measurements

**Fig. 5:**
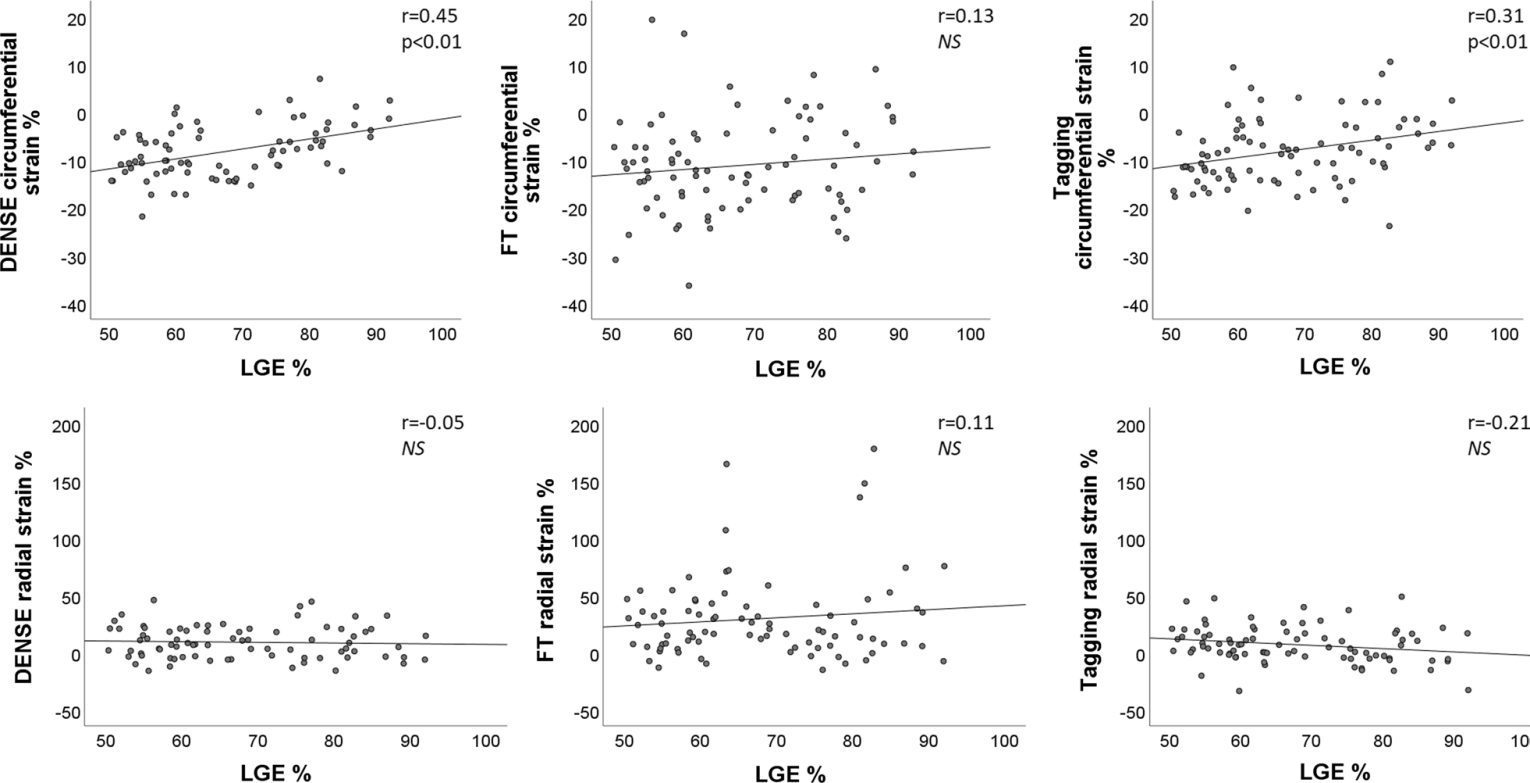
Segmental strain in segments with late gadolinium enhancement (LGE) transmurality > 50%. Relationship between segmental circumferential strain (top row) and segmental radial strain (lower row) and LGE transmurality in segments with LGE > 50% (84 segments from 34 patients). Strain was assessed by DENSE, FT and tagging. The best-fit linear regression as well as the Pearson correlation coefficient and its p-value are shown for each plot. *NS* no significant correlation

**Fig. 6:**
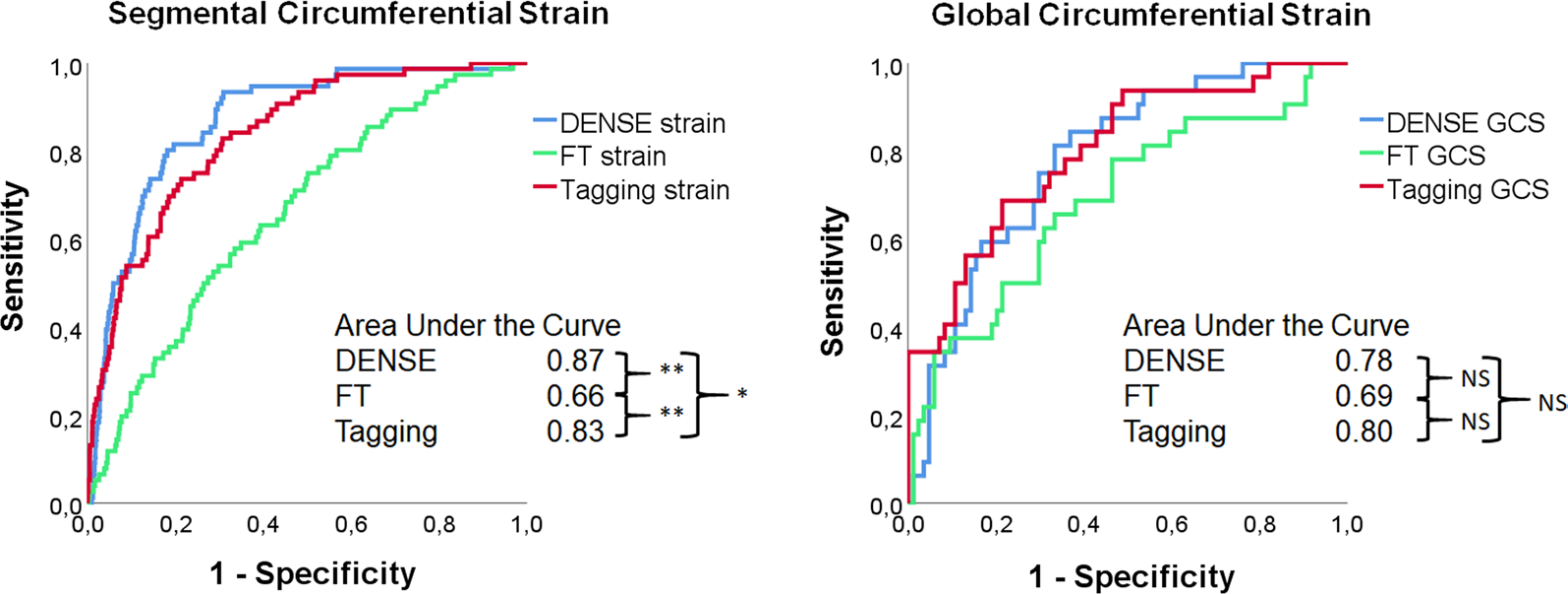
Receiver operator characteristics (ROC)-analysis of segmental and global strain. To the left ROC curves for the detection of infarcted segments with scar transmurality exceeding 50% LGE (84 [34 patients of 116] out of 1856 segments) using circumferential strain from DENSE, FT and Tagging at end systole. To the right ROC curves for the detection of infarcted segments with scar transmurality exceeding 50% LGE (34 patients out of 116 patients) using global circumferential strain (GCS) from DENSE, FT and Tagging at end systole. AUC values are shown in the lower right corner with significance level *p < 0.05 and **p < 0.01

Segmental circumferential strain assessed by DENSE, FT, and tagging showed a low but significant positive correlation with segmental scar transmurality. Based on all 1856 segments from 116 patients, the correlation was r = 0.41 for DENSE (− 15.4 ± 5.2%), r = 0.15 for FT (− 16.8 ± 10.3%), and r = 0.37 for tagging (− 16.6 ± 6.5%). Segmental radial strain displayed low negative correlations with scar transmurality, r = − 0.10 (DENSE), r = − 0.07 (FT) and r = − 0.16 (tagging). When limited to segments with a transmurality > 50% per segment (n = 84, 34 patients), the correlation for radial strain was statistically significant for DENSE (r = 0.45, p < 0.01) and tagging (r = 0.31, p < 0.01) but not for FT (Fig. 5). ROC analysis of segmental circumferential strain for the detection of scar transmurality > 50% based on all segments in Fig. 6, (left), showed the largest area under the curve (AUC) for strain amplitude from DENSE, AUC 0.87 (FT 0.66, p < 0.001 and tagging 0.83, p = 0.03).When the specificity was set to 80%, the sensitivity to detect scar transmurality > 50% was 82%, 35%, and 71% for DENSE, FT and tagging circumferential strain, respectively. In Fig. 6 (right), use of GCS for the detection of any segment with LGE > 50% in an individual patient shows AUC for DENSE 0.78, for FT 0.69 and for tagging 0.69 without significant difference between the methods.

There were slight topographic differences when segmental circumferential strain was correlated with LGE transmurality in the basal, mid and apical LV slices. In the apex (464 segments), DENSE vs LGE correlated at r = 0.59, FT at r = 0.22 and tagging at r = 0.51; in midventricular segments (696 segments) the correlation with LGE was for DENSE r = 0.36, FT r = 0.15 and tagging r = 0.36. In the basal 696 segments the correlations with LGE were for DENSE r = 0.31, FT r = 0.08 and tagging r = 0.27. Thus, segmental circumferential strain from DENSE and tagging correlated best with segmental scar transmurality in the apical part of the LV.

## Discussion

In this study of 116 patients with high risk of CAD, we showed that DENSE segmental circumferential strain has a higher AUC than tagging and FT for the detection of segments with LGE transmurality > 50%. We also show that the sensitivity at 80% specificity for using segmental circumferential strain to detect LGE transmurality > 50% was significantly higher for DENSE than for tagging and FT.

In a previous study comparing all three techniques, a fairly good agreement could be seen between all three methods when circumferential strain was measured in healthy subjects and in patients with cardiomyopathy of various etiology [21]. In our study, DENSE and tagging were clearly superior to FT to detect LGE of different severity. However, in the radial direction, segmental strain did not show a significant correlation with transmural extent of LGE, possibly because of the thin LV wall which complicated tracking. This is in contrast to the early findings of Maret et al. whose patients with scar had on average more than three times as large infarcts (17%) [7] as our group of patients with myocardial disease (5%). Their scar group was more homogeneous, and the strain measurements were averaged from three segmentations which could partly explain the difference. However, even if limited to patients with segmental extent of LGE > 50% compared to those free of myocardial disease, segmental radial strain could not differentiate between the patient groups regardless whether strain was derived from FT or tagging. It should be borne in mind though, that group averages of segmental radial strain in patients with transmurality > 50% were significantly lower than those in the healthy group, when determined with all three techniques (Fig. 2). In summary, circumferential strain correlates better than radial strain with the transmurality of LGE, in agreement with previous reports [13, 22, 23].

Our study also aimed to compare different CMR techniques of deformation measurement with clinical parameters such as LVEF and extent of LGE. In global assessment, GRS showed a low but significant correlation with LVEF and global extent of LGE when derived from DENSE, and no significant correlation when derived from FT or tagging. This indicates that radial strain from these three methods performs poorly for the determination of cardiac deformation in patients with reduced LV thickness as seen in infarction.

At a global level, GCS from DENSE, FT and tagging correlated with LVEF, blood pressure, LVESV and LGE at a similar level. A somewhat lower correlation was seen between FT and LVEDV. Other studies have shown a good agreement between FT and tagging at the global level [24, 25]. Based on these observations, we suggest that the size of the LV and the level of blood pressure at the investigation need to be accounted for when strain amplitude is determined.

Torsion from DENSE and tagging showed somewhat lower correlation with global clinical measures than strain amplitude, especially when torsion was determined from FT. The torsion values for the patients with a low probability of myocardial disease were in agreement with previously published values for DENSE [26–28] and from FT [28–31] for healthy subjects. LV torsion has been shown to be an important global parameter, but expresses only one component of the 3D strain tensor [32]. If complemented with regional and global strain amplitude, such a combined measure could possibly better reflect cardiac pumping.

All patients fulfilled the inclusion criteria, but some had few signs of myocardial disease, while others had experienced severe myocardial infarctions. Despite that variability, DENSE produced high diagnostic accuracy in all aspects tested. Since this study comprised patients and not healthy subjects, we could not determine reference values for the three methods, which we tried to circumvent in part by separately analyzing results from the relatively healthy participants with few signs of myocardial disease. We also added a group of healthy subjects which displayed values of DENSE and FT strain similar to those without manifest imaging signs of heart disease. However, healthy subjects may also differ from the general population due to a different selection bias. The study population largely reflects a CAD population with regard to age and gender [33], which makes the result of the study to some extent applicable to CAD patients in general.

DENSE and tagging share similarities in how the myocardium is tracked resulting in similar segmental strain and torsion. FT is dependent on several features that are tracked over time which makes this method sensitive to image quality as well as to through-plane motion.

## Limitations

Only Lagrangian strain was calculated for the comparison between all three methods. The use of Eulerian strain has also been reported [34], which in some situations produces results different from those obtained with Lagrangian strain [35]. Some authors have used several (most often three) FT segmentations to reduce variability [7, 15, 16], but we decided to use single measurements for each technique to avoid favoring one over the others. Some researchers propose that torsion should be normalized to the LV radius, but this was not considered necessary in this study since the three methods were applied to images obtained from the same individuals in the same scanning session. Furthermore, this version of DENSE only calculated strain and torsion in three time points while FT and tagging both cover the complete cardiac cycle. To enable this comparison, only the end systolic phase was used.

There are phantoms for deformation CMR but no consensus in terms of a general gold standard for clinically measuring strain with CMR. However, an in vivo comparison in patients with various extent of LV hypertrophy and myocardial scar offers an opportunity to compare the ability to detect reduced deformation.

All images were acquired on the same CMR scanner, but the post processing software was obtained from three separate vendors. FT software may use different technology, either variants of optical flow [36] or non-rigid elastic registration [37] which affects outcome mainly for strain amplitude in the longitudinal and radial directions [21]. The choice of post processing software was based on local availability and experience.

## Conclusions

Circumferential strain amplitude quantified using DENSE showed the highest AUC for detecting infarct scar transmurality > 50%, compared to tagging and FT. The correlation between GCS from DENSE, tagging and FT compared to global parameters such as LVEF and LGE transmural scar percentage was similar for the three strain methods. GCS was superior to torsion when compared with global measurements of volume and function.

## Data Availability

The datasets used and/or analyzed during the current study are available from the corresponding author on reasonable request.

## Abbreviations

AUC: Area under the curve
bSSFP: Balanced steady-state free precession
CAD: Coronary artery disease
CMR: Cardiovascular magnetic resonance
CSPAMM: Complementary spatial modulation of magnetization
DBP: Diastolic blood pressure
DENSE: Displacement encoding with stimulated echoes
ECG: Electrocardiogram
EF: Ejection fraction
ES: End-systole
FT: Feature tracking
GCS: Global circumferential strain
GRS: Global radial strain
Hz: Hertz
LAx: Long-axis
LGE: Late gadolinium enhancement
LV: Left ventricle/left ventricular
LVEDV: Left ventricular end-diastolic volume
LVEF: Left ventricular ejection fraction
LVESV: Left ventricular end-systolic volume
LVM: Left ventricular mass
LVMI: Left ventricular mass index
RV: Right ventricle/right ventricular
ROC: Receiver operating characteristics
SAx: Short-axis
TE: Echo time
TR: Repetition time
